# Hair Loss Associated With Glucagon-Like Peptide-1 (GLP-1) Receptor Agonist Use: A Systematic Review

**DOI:** 10.7759/cureus.92454

**Published:** 2025-09-16

**Authors:** Omar A Alsuwailem, Rawan Alanazi, Hessa M Almutairi, Rayan H Asiree, Wasan Almutairi, Taghreed M Almutairi, Alia Zamandar, Saleh Alkhames

**Affiliations:** 1 College of Medicine and Surgery, King Faisal University, Al-Ahsa, SAU; 2 College of Medicine, Dar Al Uloom University, Riyadh, SAU; 3 College of Medicine, Majmaah University, Al Majma'ah, SAU; 4 College of Medicine, Almaarefa University, Riyadh, SAU; 5 Dermatology, Security Forces Hospital, Riyadh, SAU

**Keywords:** adverse event, alopecia, glp-1 receptor agonist, hair loss, systematic review

## Abstract

Glucagon-like peptide-1 receptor agonists (GLP-1 RAs) have recently gained widespread use among patients with type 2 diabetes mellitus or obesity, owing to their substantial impact in lowering blood glucose levels and reducing body weight. Apart from this, the FDA Adverse Event Reporting System (FAERS) identified several dermatological side effects, including hair loss, associated with the administration of GLP-1 RAs, prompting further research. This systematic review aimed to provide a comprehensive and up-to-date overview of hair loss related to the use of GLP-1 RAs. The search strategy utilized PubMed, SCOPUS, and Web of Science databases using key terms: (("GLP-1 receptor agonist"[Mesh] OR "GLP1- receptor agonist"[tiab] OR "Glucagon-Like Peptide 1"[tiab] OR "GLP-1 agonist"[tiab] OR semaglutide[tiab] OR liraglutide[tiab] OR tirzepatide[tiab] OR exenatide[tiab] OR dulaglutide[tiab]) AND (hair loss[Mesh] OR alopecia[tiab] OR "telogen effluvium"[tiab] OR "alopecia areata"[tiab] OR hair[tiab]) ). Including all primary English studies, the hair loss outcomes associated with the use of GLP-1 RA in adults were reported without time restriction. A total of five relevant studies were included in this review, encompassing 2,905 adult patients who received subcutaneous trizepatide mainly on a weekly basis. The study yielded conflicting findings, with some indicating significant improvement and hair regrowth, while others reported hair loss as an adverse dermatological event. Further research is recommended to clarify the relationship between GLP-1 RAs and alopecia.

## Introduction and background

Glucagon-like peptide-1 receptor agonists (GLP-1 RAs) are antidiabetic medications that imitate the effect of the naturally occurring GLP-1. Regulating postprandial glucose secretion from the liver, delaying glucose absorption in the intestine, and promoting satiety are the primary mechanisms of action that aid in lowering blood glucose levels and reducing body weight [1,2]. Accordingly, GLP-1 RAs are FDA-approved for the treatment of type 2 diabetes mellitus (T2DM) and obesity. Examples of GLP-1 RA include dulaglutide, liraglutide, semaglutide, exenatide, and tirzepatide. They administered it orally or subcutaneously, at daily or weekly doses, depending on the indication [3].

Despite the marked glycemic control, weight loss, and cardiovascular benefits of GLP-1 RA, they are associated with several adverse effects, ranging from mild and tolerable, such as diarrhea, headache, dizziness, nausea, and vomiting, in addition to severe side effects that could lead to treatment discontinuation, including pancreatitis, acute kidney injury, and tachycardia [4]. Moreover, the literature has raised concerns about dermatological adverse events related to the use of GLP-1 RAs, including erythema, injected site pruritus, acne, hair loss, and many other adverse events [4,5]. Recently, important pharmacovigilance and safety databases like the FDA Adverse Event Reporting System (FAERS), Database of Adverse Event Notifications (DAEN), EudraVigilance, and VigiBase, implementing intensive analysis to investigate the adverse events of diabetic medications, reported that GLP-1 RAs were the most common medications causing dermatological adverse events like hair loss [6]. 

Furthermore, Tran et al. conducted a systematic review on the dermatological disorders related to the use of semaglutide, revealing some incidence of alopecia. Currently, evidence-based studies are directed at assessing the adverse events associated with GLP-1 RAs that could affect treatment adherence or patient quality of life, since this class has been widely used nowadays [7]. 

In fact, alopecia has a negative psychological and emotional impact on patients, as well as a poor quality of life. Even with the availability of several pharmacological and non-pharmacological interventions, patients find alopecia uncomfortable and restricting their normal activities [8,9]. A systematic review supports this, including more than 70 studies that found a high incidence of psychiatric disorders like depression, anxiety, and negative emotions in alopecia patients in comparison to non-alopecia patients [10]. 

To the best of our knowledge, no systematic review has focused on hair loss as an adverse event of GLP-1 RAs, defining its incidence, mechanisms, and clinical characteristics. Therefore, this study aimed to review the current evidence on hair loss associated with GLP-1 RA use, providing valuable insights for physicians and policymakers to maximize the benefits of the medication and minimize its undesirable effects.

## Review

Methods

Search Strategy

This review followed the Preferred Reporting Items for Systematic Reviews and Meta-Analyses (PRISMA) framework for systematic reviews and meta-analyses guidelines [11]. It utilized three electronic databases, namely, PubMed, SCOPUS, and Web of Science, using key terms: (("GLP-1 receptor agonist"[Mesh] OR "GLP1- receptor agonist"[tiab] OR "Glucagon-Like Peptide 1"[tiab] OR "GLP-1 agonist"[tiab] OR semaglutide[tiab] OR liraglutide[tiab] OR tirzepatide[tiab] OR exenatide[tiab] OR dulaglutide[tiab]) AND (hair loss[Mesh] OR alopecia[tiab] OR "telogen effluvium"[tiab] OR "alopecia areata"[tiab] OR hair[tiab])).

Eligibility Criteria

All primary English studies reporting the hair loss outcomes associated with the use of GLP-1 RA in adults were included without time restriction. However, secondary, non-English reviews and studies addressing any dermatological conditions other than hair loss were excluded from this review.

Data Extraction and Synthesis

Following the removal of duplicates, the titles and abstracts of the retrieved articles were revised according to the eligibility criteria, and then the remaining articles were screened for full text. The data extracted manually from the relevant studies included the first author's last name, year of publication, country of origin, study design, and study duration. In addition, population characteristics such as sample size, gender, mean age, mean body mass index (BMI) (kg/m^2^), mean weight change (kg), mean change in HbA1c (%), and duration of hair loss before the intervention (years). Moreover, the type of GLP-1 RA used, the mean duration of therapy (years), the hair loss outcome, and its type.

Quality Assessment 

The risk-of-bias assessment was implemented for each included study using the appropriate tool based on the study design. For the observational studies, the Cochrane Collaboration's Risk of Bias (ROBINS-1) tool was used, which assesses seven domains: confounding, selection bias, classification of intervention, deviation from the intended intervention, missing data, measurement of outcomes, and selection of the reported results. Based on the assessment of individual domains, the overall risk of bias for each study was judged as no information, low, moderate, serious, or critical, in addition to the ROB-2 tool, which evaluated the randomized clinical trials through main five domains: randomization bias, deviation from the intended intervention, missing outcome data, measurement of the outcomes, and selection of the reported results [12]. Accordingly, the total risk of bias was judged as some concerns, low, and high. Then, a third tool, the Joanna Briggs Institute (JBI) critical appraisal checklist, was utilized for case reports [13]. The JBI uses eight items assessing the detailed case description, diagnosis, intervention, and outcomes. Each item was evaluated using the categories: "yes," "no," "unclear," or "not applicable." Based on the percentage of criteria fulfilled, studies were classified as "good" (≥75%), "fair" (50-74%), or "poor" (<50%) [14].

Results

**Figure 1:**
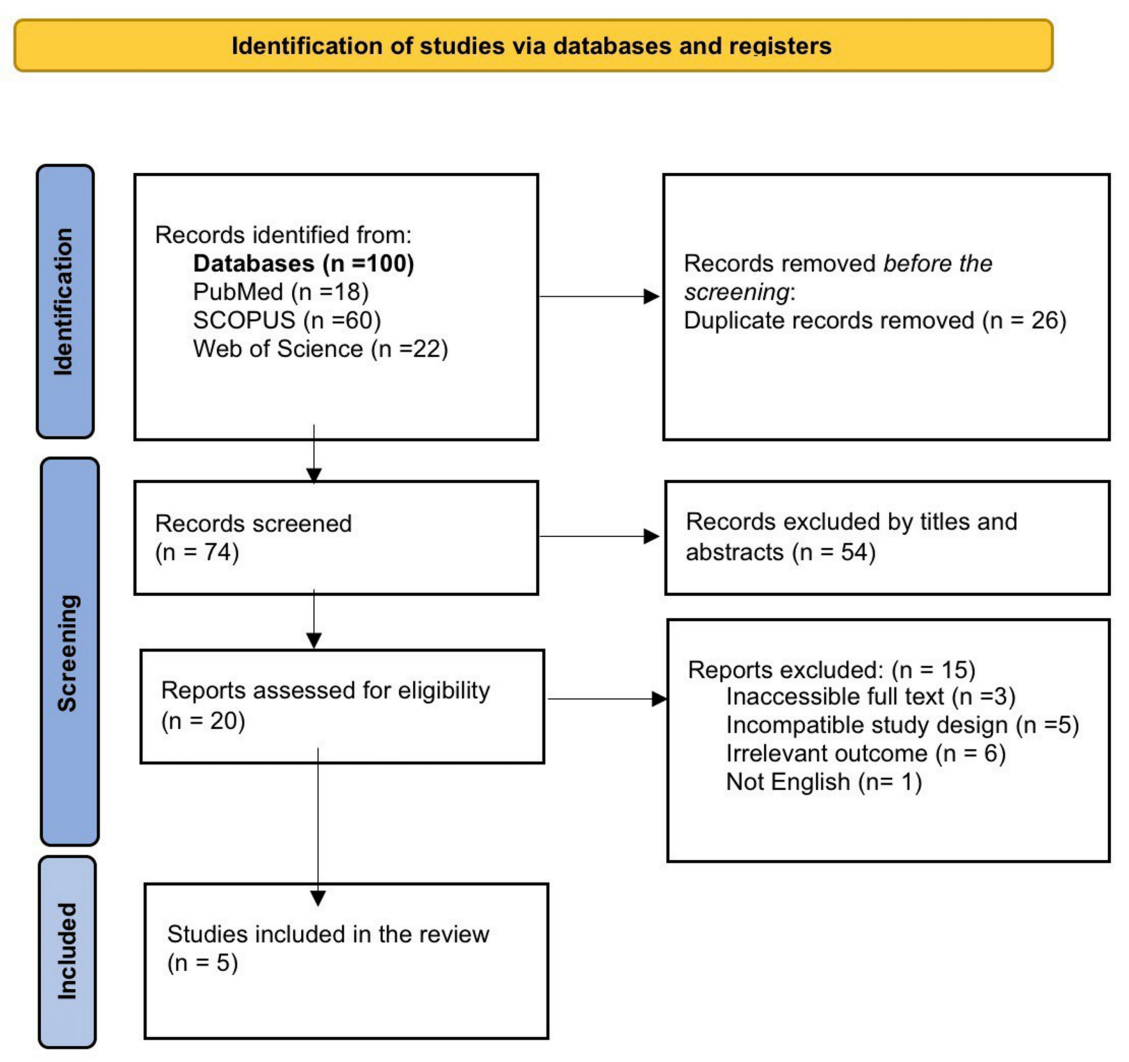
Preferred Reporting Items for Systematic Reviews and Meta-Analyses (PRISMA) flow diagram for the included studies in the systematic review

Figure 1 shows that a total of 100 articles were retrieved from the three databases; among them, 74 studies were screened after removing 26 duplicates. Following the exclusion of the irrelevant studies, only five studies were included in this review, as shown in the PRISMA diagram.

General Characteristics of the Included Studies

Table 1 illustrates a total of 2,905 adult patients included in this review, distributed across two retrospective studies in the USA, two case reports in the USA, and one multicenter, randomized controlled trial [15-19]. The mean patients' age ranged from 44.9 to 57 years, and female gender was predominant. The majority were classified as overweight or obese and had experienced hair loss for over one year before the initiation of any intervention.

**Table 1:** General characteristics of the included studies SD: standard deviation, RCT: randomized clinical trial, NA: not available, BMI: body mass index

Author, Year, Country	Study design	Sample size N (%)	Gender		Mean (SD) age (years)	Mean BMI (kg/m^2^) / category N (%)	Duration of hair loss (years)
			Female N (%)	Male N (%)			
Burke et al., 2025, USA^[15]^	Retrospective from 2021 to 2023	283	185 (65.4)	98 (34.6)	56.3 ±12.4	Normal: 15 (5.3). Overweight: 75 (26.5). Class I obesity: 92 (32.5). Class II obesity: 58 (20.5). Class III obesity: 43 (15.2%)	NA
Desir et al., 2025, USA ^[16]^	Retrospective from 2019 to 2023	Total: 81. Responder: 47 (58%). Non-responder: 34 (42%)	81 (100)	0 (0)	Responder: 47 ±11.43. Non-responder: 47.76 ± 15.03	Responder: 37.25 ± 8.79. Non-responder: 36.23 ± 7.84	<1 year Responder: 4 (8.51). Non-responder: 1 (2.94). 1-2 Responder:15 (31.9). Non-responder: 13 (38.24). 3-5 Responder: 5 (10.64). Non-responder: 3 (8.82). 6-10 Responder: 13 (27.6). Non-responder: 3 (8.82). >10 Responder: 3 (6.4). Non-responder: 3 (8.82)
Morrissette et al., 2024, USA ^[17]^	Case report	1	0 (0)	1 (100)	In 40s	N/A	30
Gordon et al., 2024, USA^[18]^	Case report	1	0 (0)	1 (100)	57	33.45	1
Jastreboff et al., 2022, multicenter ^[19]^	Phase 3 double-blind RCT, from Dec 2019 to April 2022	Total: 2539, *G1: 630, G2: 636, G3: 630 Placebo: 643	1714 (67.5)	825 (32.5)	44.9± 12.5	38± 6.8 <30: 140 (5.5) ≥30 to <35:876 (34.5) ≥35 to <40:720 (28.4) ≥40: 803 (31.6)	N/A

Hair Loss Outcome Associated With GLP-1 RA Use

Table 2 demonstrates that subcutaneous tirzepatide was the most commonly used GLP-1 RA among the studies. Weight loss was observed in all studies following the use of GLP-1 RA, with a mean therapy duration ranging from 0.75 to three years. In addition, one study reported a significant reduction, while others showed an elevation in the HbA1c [16,18]. Regarding hair loss, several types were mentioned, such as androgenic alopecia, alopecia, telogen effluvium, central centrifugal cicatricial alopecia, and folliculitis decalvans. Furthermore, three studies highlighted a significant improvement and hair regrowth in the affected area after the use of weekly subcutaneous 2.5 mg tirzepatide, which was then titrated to 7.5 mg [16-18]. On the other hand, two studies reported hair loss as an adverse dermatological event related to semaglutide and tirzepatide therapies [15,19].

**Table 2:** Hair loss outcome associated with GLP-1 RA use *Change between the initial diagnosis visit and the follow-up visit. ** G1: Tirzepatide 5 mg, G2: Tirzepatide 10 mg, G3: Tirzepatide 15 mg SD: standard deviation, N/A: not available, GLP-1 RA: glucagon-like peptide-1 receptor agonist

Author, year, country	GLP-1 RA used N (%)	Mean (SD) duration of therapy (years)	Mean (SD) weight change* (kg)	Mean (SD) HbA1c (%) change*	Hair loss		Type of hair loss N (%)	Conclusion
					Yes N (%)	No N (%)		
Burke et al., 2025, USA ^^[15]^^	Dulaglutide: 62 (21.9) Liraglutide: 33 (11.6) Semaglutide: 24 (82.7) Tirzepatide: 130 (45.9)	NA	-6.2% ±8.3 Weight loss 213 (76.9%) Weight gain 49 (5.4%) No change 15 (17.7%)	N/A	35 (14.2) Worsening 29 (11.9) Resolved 1 (0.4) Stabilized 2 (0.8)	207 (84.1)	Androgenic alopecia 19 (7.7) Telogen effluvium 10 (4.1) Unspecified hair loss 3 (1.2) Unspecified alopecia 4 (1.6) Others 6 (2.4)	Semaglutide was responsible for (OR: 6.97) hair loss. In comparison, tirzepatide was weakly associated (p=0.054) with telogen effluvium.
Desir et al., 2025, USA ^^[16]^^	N/A	Responder: 3.04 ±2.61 Non-responder: 2.42 ± 2.8 (P=0.04)	Responder: -3.76 ±13.18 Non-responder: -5.02 ±11.21	Responder: -0.47± 0.74 Non-responder: -0.1 ±0.23 (P=0.01)	N/A		Central centrifugal cicatricial alopecia	GLP-1 RA with extended therapy duration demonstrated a significant improvement in the metabolic profile, thereby promoting hair regrowth.
Morrissette et al., 2024, USA ^^[17]^^	Subcutaneous tirzepatide (2.5à12.5à7.5 mg weekly)	0.75	-22.7	N/A	1 (100)		Folliculitis decalvans	Tirzepatide made a substantial improvement in the FD by inducing hair regrowth in the affected area.
Gordon et al., 2024, USA ^^[18]^^	Subcutaneous tirzepatide (2.5à5à7.5 mg weekly)	1	-22.7	+0.5	1 (100)		Androgenic alopecia	The patient experienced significant hair regrowth and an increase in hair density after one year of treatment.
Jastreboff et al., 2022, multicenter ^^[19]^^	Tirzepatide (5, 10, and 15 mg weekly)	1.4	**G1: -15 G2: -19.5 G3: -20.9 Placebo: -3.1	NA	G1:32 (5.1) G2: 34 (5.3) G3: 31 (4.9) , Placebo: 6 (0.9)		Alopecia	Different doses of subcutaneous Tirzepatide were associated with several adverse events, including alopecia, compared to the placebo.

Quality Assessment

**Figure 2:**
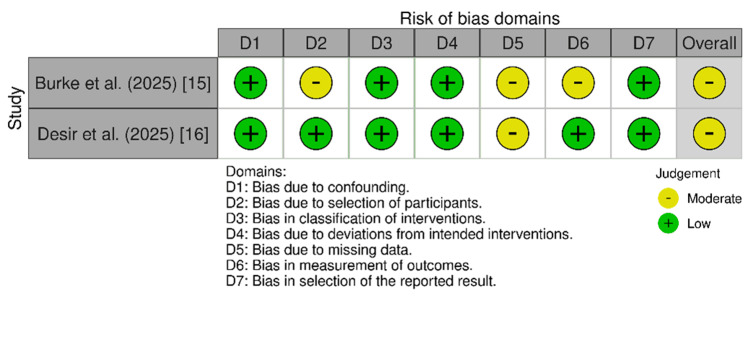
Risk-of-bias assessment using the ROBINS-I tool for the relevant studies (n = 2)

**Figure 3:**
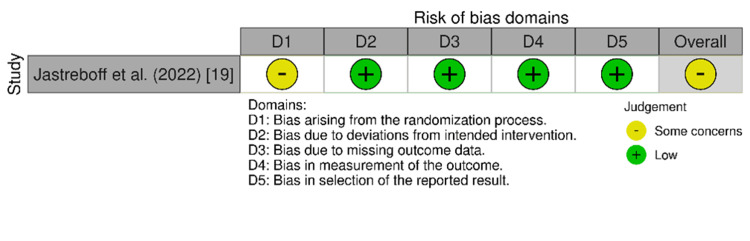
Risk-of-bias assessment using the ROB-2 tool for the relevant study (n = 1)

The risk-of-bias assessment of the two observational studies was conducted using the Cochrane Collaboration's ROBINS-1 tool, which revealed a moderate risk in both studies [15,16]. The evaluation of the RCT by the ROB-2 tool showed some concerns about risk of bias [19]. Figures 2-3 show the results of the ROBINS-I and ROB-2 quality assessments, which were created with Robvis (risk-of-bias VISualization). In addition, as shown in Table 3, using the JBI checklist, one case report was of good quality, while the other had fair quality [17,18].

**Table 3:** Risk-of-bias assessment using the Joanna-Briggs Institute (JBI) critical appraisal checklist for the relevant studies (n = 2)

Study (author, year)	Q1	Q2	Q3	Q4	Q5	Q6	Q7	Q8	Overall risk
Morrissette et al. (2024) [17]	❌	❌	✔	❌	✔	✔	❌	✔	Fair
Gordon et al. (2024) [18]	✔	❌	✔	✔	✔	✔	✔	✔	Good

Discussion

Coinciding with the rapid widespread use of GLP-1 RAs, as a result of their ability to improve the health outcomes of obese or diabetic patients, several adverse events have been recently recorded. In addition, the clinical evidence on hair loss specifically is minimal, which could be attributed to underreporting. Therefore, a comprehensive overview of the updated studies addressing the consequences of hair loss from GLP-1 RAs could add insightful value to the clinical research body [7]. This review presented the latest studies on hair loss associated with GLP-1 RAs.

Our findings appeared to be controversial, as some studies suggested that tirzepatide may have a potential positive effect on improving hair regrowth. By contrast, others established a hair loss pattern, such as androgenic alopecia or telogen effluvium, related to the use of semaglutide and tirzepatide, aligning with the results of Desai et al. [20].

In particular, List et al. explained the mechanism of GLP-1 RAs in enhancing hair growth through the expression of GLP-1 receptors around the hair follicles, thus activating the MAPK/ERK signalling pathway, which results in cellular proliferation and differentiation required for hair follicle growth and maintenance [21]. Another theory suggests that achieving good glycemic control and reducing body weight can improve blood flow and insulin sensitivity [18].

By contrast, Tran et al. observed that the administration of different dosages of oral semaglutide was significantly associated with a high incidence of hair loss (aROR: 1.42, 95% CI 1.3-1.55) [7]. Similarly, Godfrey et al. conducted disproportionality analysis using the FAERS, indicating a marked relation between semaglutide and tirzepatide with alopecia (ROR: 2.46, 95% CI: 2.14-2.83, ROR: 1.73, 95% CI: 1.42-2.09, respectively). However, the remaining GLP-1 RAs failed to show any significant association with alopecia [22]. Likewise, Moll et al. reported a relative risk of 5.67 (95% CI: 2.47-13) for alopecia associated with tirzepatide [23].

Nonetheless, the degree of body weight reduction is found to influence the severity of hair loss directly. The Wegovy clinical trial on semaglutide demonstrated that 5.3% of alopecia cases observed in patients had a greater than 20% body weight reduction, while 2.5% of those losing less than 20% of their body weight experienced alopecia [24].

Although the proposed mechanism by which GLP-1 RAs cause alopecia is still not well established. Some studies describe it as rapid weight loss leading to malnutrition and essential vitamin deficiencies, which disrupts hair growth and triggers the development of alopecia [25].

Moreover, it is documented that semaglutide has been thought to cause hormonal changes that raise the chance of androgenic alopecia and having thin hair, which could be persistent even after medication discontinuation [6].

Unlike the gastrointestinal side effects associated with the use of GLP-1 RAs, which often improve over time, alopecia or hair loss can progress alongside the treatment journey. Consequently, patients could seek treatment discontinuation or non-adherence. Thomsen et al. found that diabetic or obese patients tend to quit their therapy after the first year because of adverse events and economic burden [26]. This results in a financial burden on the healthcare system due to the rise in hospitalizations, comorbidities, and mortality rates [27]. Therefore, pharmacovigilance programs must shed light on hair loss as an emerging adverse event in patients undergoing GLP-1 RA therapy. In addition, research should focus on developing updated clinical guidelines that take into consideration the early and proper management of less recognized side effects to achieve better patient health outcomes.

Limitations

Since hair loss as an adverse event to the use of GLP-1 RAs is a novel topic and remains under investigation in the literature, only a small number of studies were included in this review. Another limitation of this research is gender bias, as most of the patients were female. In addition, the studies demonstrated a study design, population characteristics, and outcome variability, thus limiting the ability to conduct a quantitative synthesis. In addition to the inclusion of brief reports with restricted data presentation, this approach could be beneficial as early documentation for new adverse events and provide important insights for future research.

## Conclusions

Our review yielded conflicting findings, which emphasize the need for additional intensive future research to elucidate the connection between GLP-1 RAs and alopecia, taking into consideration the different types of GLP-1 RAs, administration routes, and dosages. In addition, longitudinal studies, as well as clinical trials with large sample sizes, could provide robust data on the metabolic changes caused by these drugs, thereby predicting their impact on hair loss. In addition, it is recommended that healthcare professionals focus on and document the incidence of hair loss associated with GLP-1 RAs for better patient medication compliance. Lastly, we suggest adding a patient education statement recommending that clinicians counsel patients about the potential risk of hair loss and encourage them to report any such occurrences.
